# What approaches to social prescribing work, for whom, and in what circumstances? A realist review

**DOI:** 10.1111/hsc.12839

**Published:** 2019-09-09

**Authors:** Kerryn Husk, Kelly Blockley, Rebecca Lovell, Alison Bethel, Iain Lang, Richard Byng, Ruth Garside

**Affiliations:** ^1^ NIHR CLAHRC South West Peninsula (PenCLAHRC) Faculty of Medicine and Dentistry University of Plymouth Plymouth UK; ^2^ NIHR CLAHRC South West Peninsula (PenCLAHRC) College of Medicine and Health University of Exeter Exeter UK; ^3^ European Centre for Environment and Human Health College of Medicine and Health University of Exeter Exeter UK

**Keywords:** health services research, primary care, social and health services

## Abstract

The use of non‐medical referral, community referral or social prescribing interventions has been proposed as a cost‐effective alternative to help those with long‐term conditions manage their illness and improve health and well‐being. However, the evidence base for social prescribing currently lags considerably behind practice. In this paper, we explore what is known about whether different methods of social prescribing referral and supported uptake do (or do not) work. Supported by an Expert Advisory Group, we conducted a realist review in two phases. The first identified evidence specifically relating to social prescribing in order to develop programme theories in the form of ‘if‐then’ statements, articulating how social prescribing models are expected to work. In the second phase, we aimed to clarify these processes and include broader evidence to better explain the proposed mechanisms. The first phase resulted in 109 studies contributing to the synthesis, and the second phase 34. We generated 40 statements relating to organising principles of how the referral takes place (Enrolment), is accepted (Engagement), and completing an activity (Adherence). Six of these statements were prioritised using web‐based nominal group technique by our Expert Group. Studies indicate that patients are more likely to enrol if they believe the social prescription will be of benefit, the referral is presented in an acceptable way that matches their needs and expectations, and concerns elicited and addressed appropriately by the referrer. Patients are more likely to engage if the activity is both accessible and transit to the first session supported. Adherence to activity programmes can be impacted through having an activity leader who is skilled and knowledgeable or through changes in the patient's conditions or symptoms. However, the evidence base is not sufficiently developed methodologically for us to make any general inferences about effectiveness of particular models or approaches.

**What is known about this topic?**

- Social prescribing is gaining popularity in the UK
- The evidence base for social prescribing approaches lags behind practice and roll‐out
- Given a rapid planned expansion of programmes, there is a need to understand what works, for whom, in what ways.

**What this paper adds**

- Social prescribing is not a single intervention but a pathway and series of relationships, all of which need to function to meet patient need
- The role of the link worker is key to avoid the process being disrupted
- Multiple interacting factors at three key stages (our organising principles: enrolment, engagement and adherence) contribute to pathway ‘success’

## 1 INTRODUCTION

The prescribing of non‐medical, community or social activities is becoming more common in England as an option to help people manage and prevent illness and improve their health and well‐being (Loftus, McCauley, & McCarron, 2017; Pilkington, Loef, & Polley, 2017). These approaches, often labelled ‘social prescribing’, can range from financial advice to walking groups and enable healthcare providers to respond to a broad range of patient needs, as well as potentially reducing GP and emergency department service demand (Polley, Bertotti, Kimberlee, Pilkington, & Refsum, 2017). Social prescribing models provide more tools to incorporate the social as a cause of ill health and facilitates opportunities for patient contact with non‐medical professionals, treatments and activities. The current UK Secretary of State for Health and Social Care, Matt Hancock, has stated that social prescribing is a priority and will be available in every GP practice by 2024 (Hancock, 2018). The newly published NHS England Long Term Plan will fund social prescribing link workers in every newly created Primary Care Network, stating that ‘within five years over 2.5 million more people will benefit from social prescribing’ (NHS England, 2018).

Despite this proliferation the evidence base is patchy (Wilson & Booth, 2015), limited in quality and extent (Polley et al., 2017), with little consensus around appropriate outcome measures (Rempel, Wilson, Durrant, & Barnett, 2017). Previous studies highlight evidence gaps regarding effectiveness of programmes (Bickerdike, Booth, Wilson, Farley, & Wright, 2017; Chatterjee, Camic, Lockyer, & Thomson, 2017; Pescheny, Pappas, & Randhawa, 2018), the process of referral and delivery (Bickerdike et al., 2017), the suitability of the process for different health conditions (Pilkington et al., 2017), cost‐effectiveness (Bickerdike et al., 2017; Polley et al., 2017) or impact on GP workload (Loftus et al., 2017).

There is a need for further evidence regarding what constitutes good social prescribing practice and *process*, particularly given the plurality of delivery approaches, prescribed activities, and patient groups for which they are being used.

**Figure 1:**
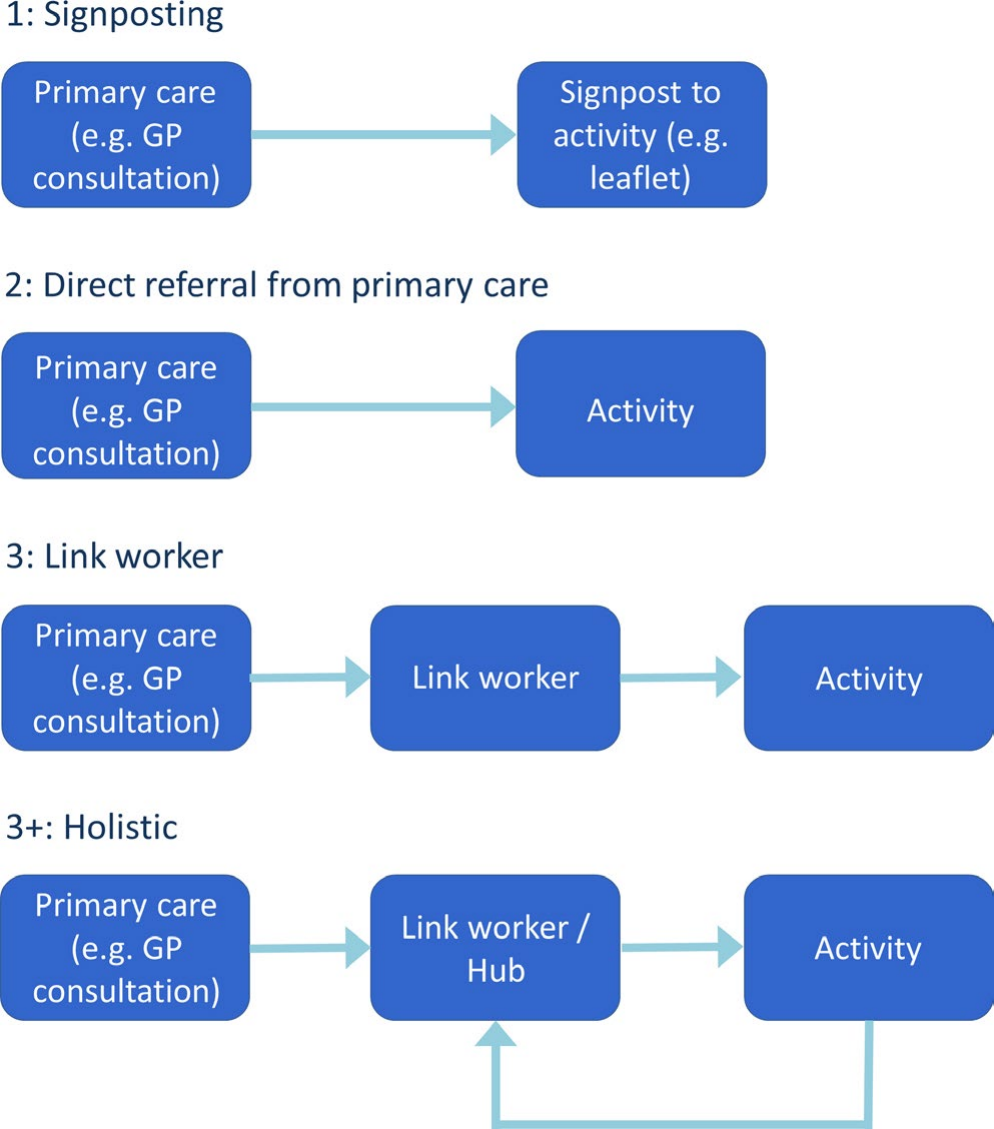
Models of social prescribing [Colour figure can be viewed at http://www.wileyonlinelibrary.com/]

In this paper, we conceptualise ‘social prescribing’ as the patient pathway from primary care to whichever activity undertaken, and that pathway can take multiple forms. Figure 1 is a simplified illustration of the main types of pathways. Importantly, government policy now supports link worker‐based (3/3+) models and will reimburse newly formed Primary Care Networks for one link worker per 30,000–50,000 population (NHS England, 2018).

The objectives of this review were to explore what is known about whether different methods of social prescribing referral and supported uptake do (or do not) work. We assert there are three key elements of social prescribing which determine success, which we use as organising principles: the successful initial referral of patients, participants attending the first activity session, and to maintain this participation over time.

## 2 METHODS

We undertook a realist review (Pawson, Greenhalgh, Harvey, & Walshe, 2005; Wong, Greenhalgh, Westhorp, Buckingham, & Pawson, 2013) as we were seeking to explicate the ways in which the process of social prescribing works, for whom, and in what circumstances. Our methodological approach is set out in detail in the published protocol (Husk et al., 2016), and definitions for methodological concepts set out in Appendix S1. The review was supported by an Expert Advisory Group, consisting of 11 individuals with experience of the creation and delivery of social prescribing models or in realist methodologies. The review had two phases: in the first phase, we identified evidence specifically relating to social prescribing to develop programme theories in the form of ‘if‐then’ statements, articulating *how* social prescribing models are expected to work. In the second phase, we aimed to clarify these processes and include broader evidence (i.e. not necessarily related to social prescribing) to better explain the proposed mechanisms.

### 2.1 Searches

We conducted two main stages of searches relating to the two phases of the review. Both searches were led by an information specialist (AB) in consultation with the review team and our Expert Advisory Group.

#### 2.1.1 First phase searches (a)

The first phase searches aimed to identify literature relating specifically to social prescribing and so we used no synonyms. The search strategy, databases and dates of searches are available in Appendix S2. Given that a great deal of the social prescribing literature is unpublished we also conducted extensive grey literature searches (Cooper, Lovell, Husk, Booth, & Garside, 2018). Initially, we contacted our expert advisory group to identify studies, individuals and organisations. We hand‐searched organisational websites (see Appendix S3) and contacted individuals by telephone. We conducted searches of grey literature databases and Google. Files containing exported results of searches were uploaded and de‐duplicated using EndNote X8.

#### 2.1.2 Second phase searches (b)

The second phase searches aimed to provide better explanations of programme theories identified and prioritised by our Expert Advisory Group. We conducted searches in MEDLINE relating to specific concepts in each theory (see Appendix S4).

### 2.2 Study selection

#### 2.2.1 Study selection from first phase searches (a)

##### Inclusion criteria

As we were looking to understand how different models of social prescribing were being used and in what ways, we included any type of article (‘article’ is defined in Appendix S1):

- Population: Any
- Intervention: Studies focusing on the transfer between primary care and community‐based activities
- Comparator: All relevant comparators such as treatment as usual, or referral to NHS services
- Outcome: Any
- Study design: We included both empirical and non‐empirical, quantitative and qualitative studies

Study selection for the first phase comprised two stages: first, two reviewers (KH and KB) independently screened titles and abstracts and, where studies appeared to meet inclusion criteria, full texts were obtained. Second, full texts were screened in the same manner. Disagreements between the two reviewers were resolved through discussion and, where needed, a third reviewer (RG). We piloted screening on a subset of articles and the inclusion criteria refined through discussion. Articles identified through grey searches were screened at full‐text.

#### 2.2.2 Study selection from second phase searches (b)

At this stage, we prioritised higher order evidence (RCTs/SRs) but included other forms of evidence as appropriate. These targeted search results were screened at full‐text by one reviewer (KH or BL) and potentially includable studies discussed by the team.

### 2.3 Data extraction

Data extraction was iterative and formed part of the analysis and aided synthesis. A coding frame was developed through discussion around our pre‐defined (Husk et al., 2016) organising principles:

1. Primary care professionals’ awareness of, and willingness to offer a social prescription and patients’ consideration of and acceptance of the prescription (Enrolment);
2. Patients’ initial participation in the activity (Engagement); and
3. Patients’ ongoing involvement with and/or uptake of prescribed activity (Adherence).

Articles were coded and contributing themes identified through an understanding of components using NVivo and data iteratively extracted against component categories.

We extracted data relating to the process by which individuals use services, the outputs of those services (e.g. the number of people moving through each stage) and, where reported, outcomes (improvements in physical or mental health).

Due to the large number of studies identified in the first phase, we used an assessment tool (Pearson et al., 2015), Appendix S1, to select studies based on their rigour and relevance for theory development, concentrating on those rated as ‘conceptually rich’. We organised and annotated studies in NVivo 11 (QSR International Pty Ltd., 2012).

For articles identified in the second phase we extracted data to better explain contexts, mechanisms and outcomes identified in the first phase. The disparate nature of these mechanisms meant that no single approach to data extraction was appropriate, rather elements relevant to the theory were noted, discussed and integrated into the analysis.

### 2.4 Analysis and synthesis

Our analysis iteratively examined data, ordered with the coding framework described above, using realist logic at two levels (Pearson et al., 2015):

- Making sense of how programmes work and the contexts in which mechanisms fire (expressed using ‘if‐then’ statements);
- Deeper explanations of these patterns using context‐mechanism‐outcome level logic.

The first stage of the analytic process consisted of the identification of prominent recurring patterns (demi‐regularities) and their explanation using ‘if X‐then Y’ structured statements. This was initially based around our three organising principles (Enrolment, Engagement and Adherence), later refined into four subcategories (patient, GP, process and activity). The team met and iteratively reconsidered initial statements in the light of new data and, where necessary, refined them accordingly. This process resulted in 40 statements of how social prescriptions operate, structured around the 12 areas (three organising principles, each then broken down into four subcategories).

In the second stage of analysis, we aimed to develop explanations as to how enrolment, engagement and adherence were achieved, using the results of our phase two searches to interrogate contexts, mechanisms and outcomes. We were unable to do this robustly for every one of the 40 included statements and so undertook a web‐based nominal group technique prioritisation (Murphy et al., 1998; Silicon Fareast, 2006) with our Expert Advisory Group, selecting six statements to analyse in‐depth.

## 3 RESULTS

### 3.1 Search results and study characteristics

**Figure 2:**
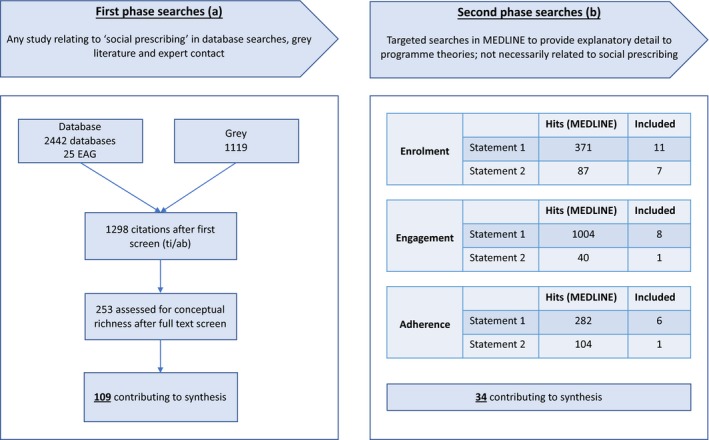
Flow diagram of included studies [Colour figure can be viewed at http://www.wileyonlinelibrary.com/]

The first phase of searching (a) resulted in a total of 3,586 hits, of which 109 contributed to the synthesis. The second phase of searching (b) resulted in a total of 1888 hits, of which 34 contributed to the synthesis. See Figure 2:

The tables below summarise included evidence according to intervention model, study design, participants and social prescribing process model (Tables 1 and 2).

**Table 1:** Included studies by intervention model, study design and included participants

	Study design											Participants						
	Primary studies					Secondary data				Other								
	Controlled studies*	Uncontrolled before and after	Survey	Qualitative	Process evaluation	Systematic Review	Secondary data analysis (registers etc.)	Lit review	Opinion	Other		Older people	Children/YP	Probation	General population**	Mental Health	Professionals	
Intervention model																		
Exercise	7	6	12	13	2	9	6	1	6	3	65	3	0	0	60	1	1	65
Green prescription	0	0	2	4	0	0	0	1	1	0	8	0	0	0	4	4	0	8
Arts on prescription	0	1	0	3	0	0	0	0	1	0	5	1	0	0	1	3	0	5
Other/generic SP	1	4	5	6	0	0	1	5	7	2	31	0	0	0	20	10	1	31
Total	8	11	19	26	2	9	7	7	15	5	**109**	4	0	0	85	18	2	**109**

Note

* Two of these are protocols.

** General population includes those with a diagnosis of CVD or type 2 diabetes.

Bold values indicate totals and are to indicate the total number of studies in the review.

**Table 2:** Included studies by intervention model and process model

		Process Model					
		1	2	3	3+	Not applicable/reported	Total
Intervention model	Exercise	1	50	4	0	11	66
	Green prescription	1	3	0	0	3	7
	Arts on prescription	0	4	0	0	1	5
	Other/generic SP	2	2	5	8	14	32
	Total	4	59	10	8	29	109

Note

Process model key: 1 = Signposting/information prescription; 2 = Primary care—activity; 3 = Primary care—link worker—activity; 3+ = Holistic process—flexible, iterative and patient‐led.

Not applicable/reported = Process not reported in paper OR reference was a more general overview of studies, for example, systematic review, commentary and description.

Unsurprisingly given its longer establishment, the majority of the evidence related to exercise programmes. Most studies included the general population or those reporting mental health problems rather than specific conditions, and were survey based or qualitative in approach.

### 3.2 Analysis and synthesis (i)—making sense of programmes

Our 40 statements of programme theories relating to the social prescription process are presented in their entirety in Appendix S5. These are the prominent recurrent patterns of if and how the referral takes place (Enrolment), is accepted (Engagement), and maintained activity (Adherence).

### 3.3 Analysis and synthesis (ii)—deeper explanations of prioritised theories

Two statements relating to each outcome were prioritised by our expert group (Table 3) for further investigation and to strengthen inferential explanations. Quotes used are summarised in Appendix S6.

**Table 3:** Prioritised programme theory statements

Enrolment	IF the patient believes the social prescribing will do them good THEN they may be receptive
	IF the referral is presented in an acceptable way and matches patient needs and expectations THEN they may be receptive
Engagement	IF the activity is accessible to the patient THEN they are more likely to attend
	IF the transit to first session is supported THEN the patient may be more likely to attend
Adherence	IF the activity leader(s) is/are skilled THEN the patient is more likely to maintain Adherence
	IF there is a significant change in patient condition or symptoms THEN this may affect Adherence

### 3.4 Enrolment (agreeing to the referral)

#### 3.4.1 Enrolment statement 1: IF the patient believes the social prescribing will do them good THEN they may be receptive and enrol

Twenty‐four studies^1^ provided information relating to this statement, and from these we identified three themes that contribute to our understanding of how the conditions of the statement are being met.

The first, expectations of consultations and solution seeking, highlighted participant motivations underpinning their visit to the GP and the need for something ‘else’ where existing options might not be working satisfactorily. For example, the quote below illustrates the acceptance of a referral that the participant sees as meeting both medical (weight, diabetes) and also personal (feeling down) needs:

I was pleased. I was struggling to control my diabetes and I thought this would help. I was feeling really down and [my GP] suggested this so I could lose weight and do something for me (ERS Research & Consultancy, 2013).

The second theme related to patients’ belief that they had a condition that the social prescription would address. In whatever way the prescription was presented, the participant should feel that their condition or symptoms will be addressed by accepting, highlighting the importance of patients’ agency in the decision. For example, the participant below believes that the referral will help them deal with their diabetes, and has navigated both a practice nurse and GP:

…the practice nurse at West Road referred me straight away so I could start to deal with it…It was my idea…I wanted to do something that would better help me get better and control the pain… (ERS Research & Consultancy, 2013)

The Health Belief Model (Mills, 2008) understands these behaviours as rational responses to a perception of illness and the evaluation of options to alleviate, which is related to motivation:

A…major theme…was the persistence with which people sought solutions to their problems, often despite formidable psychological, social and/or material obstacles. (Popay, Kowarzik, Mallinson, Mackian, & Barker, 2007)

The third theme focused on potential participants’ perception of the reliability of the provider of the activity itself. ‘Reliability’ in this context took multiple forms, but the concerns related to whether patients believed the group had adequate facilities to manage complex clients (in terms of experience and practical environment for dealing with particular symptoms and characteristics of conditions), as well as whether staff were sufficiently trained:

For participants, the most common barriers were concerns regarding staff training or appropriate facilities to manage complex patients. (Adsett, Hickey, Nagle, & Mudge, 2013)

In broadening our search for evidence (Appendix S4), we located studies describing participants’ belief in a referral being a key element of agreement between clinician and patient, making this a plausible pathway. Studies also noted that elements relating to participant belief are overlooked in consultations (Alexander et al., 2011), with self‐efficacy (Aljasem, Peyrot, Wissow, & Rubin, 2001), and a belief in relevance of the activity (Beaulieu, Beland, Roy, Falardeau, & Hebert, 1996; Bos‐Touwen, Trappenburg, van der Wulp, Schuurmans, & de Wit, 2017) important. Also noted was a disconnect between what the patient wanted in consultation and clinician understandings (Diamond & Markham, 2009; Himmel, Lippert‐Urbanke, & Kochen, 1997), which should be aligned.

#### 3.4.2 Enrolment statement 2: If the referral is presented in an acceptable way and matches patient needs and expectations THEN they may be receptive and enrol

The second statement relating to Enrolment was identified in 24 studies^2^ which contribute to our understanding of how the conditions of the statement might be met, further split into four distinct themes.

First, the specifics on the particular activity on offer were felt to be important to patients’ receptiveness, with reports of a fear of the unknown or elements of activities being challenging. An example of a mechanism to ensure fear of the unknown is overcome might be a printed resource:

…it's quite daunting coming into the leisure centre for the first time, they're not too sure what they are going to be doing…so we are trying to design a leaflet now which we are going to put out…saying exactly what they are required to do. (Moore, Moore, & Murphy, 2011).

Referrers had a role to play in allaying fears of the offer specifics as they arose:

Initial consultations were often cited as an opportunity to reassure patients that…[they] would not be expected to do anything that they were not confident about doing or which made them uncomfortable. (Moore et al., 2011)

The second theme related to the social prescribing referral process itself, where the power relationship meant advice could range from a friendly suggestion to a direct order, depending on the individual involved:

…a social prescription may be accepted by a patient just because it has the credibility of being the doctor's suggestion. (Brandling, Howitt, & Sansom, 2011)

Different groups understood a similar message from a referrer in different ways, with important cultural differences:

Many Dutch experienced the advice as being ‘just a recommendation’ [study author interpretation], which meant it was not experienced as a deciding factor…many migrant participants, however, experienced the GP as someone ‘who knows better’ and participated in the intervention because they were told to do so. (Schmidt, Absalah, Nierkens, & Stronks, 2008)

The differential and power balance had implications for practice:

So I had to change my consultation style to enable me to open up a discussion about social prescribing and if the patient was interested. (GP participant; (Friedli, Themessl‐Huber, & Butchart, 2012))

The third theme reflected what is known more broadly in healthcare but was raised in the context of social prescriptions, that the format and delivery of that referral, or the ‘thing’ that was offered to patients, varied considerably; from a formal hard‐copy:

…referral forms were provided to all…and completed on behalf of interested participants. (Adsett et al., 2013)

Through to an informal discussion:

If an individual was considered to meet the referral criteria, the project was discussed with them. (Baker & Irving, 2016)

It was reported that diversity in format and delivery of the referral affected receptiveness of the patient, though it was unclear which methods were better received than others, but rather:

Referrers should be made aware that the interactions during referral have a strong contributing effect on whether patients engage with the service offered (Brandling & House, 2009)

The last theme identified related to the symptoms that the patient presented with; how symptoms might be alleviated through the appropriate prescription. This theme relates specifically to how the referral process and presentation relates to symptoms, and the acceptability of a referral. For example, a participant discussed a GP who had identified and was addressing areas that might not be things relevant to a GP consultation:

So we talked through my situation and she wrote down the topics that I particularly wanted to be helped with. And I was really pleased to be able to have this attention, because sometimes you just don't know who to go to to ask these things, you know…And they weren't particularly things that GPs would necessarily…you know, that you would necessarily, sort of, bother them with, if you like… (Participant; (Callaghan, Shenton, Maramba, & Lloyd, 2016))

It was also important to participants that the discussion included potential risks and their mitigation:

Commonly patients…are fearful that engaging in physical activity will exacerbate their condition; similarly older individuals are often fearful of getting injured (Stirrat, 2014)

Evidence from outside the social prescribing literature reinforced these findings; a written script can contribute to the acceptance of a referral, that there are multiple ways in which instructions can be interpreted in the consultation environment (Dempster, Wildman, & Duby, 2015), and that interpretation can be culturally dependent. Ellis et al. (2015) reported attendance was impacted by method of referral in that an invitation letter was deemed ineffective and not worthy of remembering, let alone inciting action. Himmel et al. (1997) showed that nearly half of patients expected a written script yet only 40% were recognised as expecting this by their GP, implying that not only is the method itself important but also is the recognition of that desire in a consultation. Culturally, two studies (Garrett et al., 2012; Hudson et al., 2016) noted a disconnect between traditional referral offers and the British South Asian population, which led to alternative techniques being employed such as information sharing events to discuss best practice and treatment options.

### 3.5 Engagement (attending at least the first session)

#### 3.5.1 Engagement statement 1: IF the activity is accessible to the patient THEN they are more likely to attend

The first statement relating to Engagement was located in 28 studies^3^ which contribute to our understanding of how accessibility of the activity impacts on engagement, these factors were grouped into four distinct themes.

The first theme is important in considering the relationship between referrers and providers of social prescriptions, and relates to the cost of attending a social prescribing activity, which could be incurred as part of a fee for joining a group, per session, travel, or equipment needed to attend. It is possible that a modest cost would be seen as a motivating factor for attendance:

…cost[s]…were seen as advantages and disadvantages of the community‐based program, depending on individual circumstances (Adsett et al., 2013)

The second theme centred on a participant's physical proximity to the offered social prescribing activity, or if it was sufficiently close to be *perceived* as accessible, which would differ depending on car ownership status, or rural or urban location. The issue of proximity was closely related to the feeling of ‘safety’ in attending a social prescribing activity, where travel to and from locations may be seen as threatening:

the neighbourhood setting was given as a reason…participants do not feel safe in their neighbourhoods…and this was a reason to stay home (Schmidt et al., 2008)

Practicality was echoed in the third theme, the time of day that an activity was offered. Activities were offered on weekdays, morning or afternoon, evenings or weekends, with timings designed to attract different cohorts. There were unintended consequences of these timings, with some reporting negative feelings related to, for example, ‘seasonal changes in lighting’ (Stirrat, 2014).

Perception of accessibility was also impacted by our final theme, the safety, provision and availability of transport to and from the social prescribing activity offered:

[the most] …valuable form of support…was transport to appointments (Callaghan et al., 2016)

Unsurprisingly, but raised as important in the social prescribing context by study authors, this availability impacted upon acceptance:

…the only significant correlates of uptake…were car ownership and deprivation (Campbell et al., 2015)

As previously, we located broader evidence to deepen our understanding of how access might be linked to attendance in health interventions (Appendix S4). Foster and Giles‐Corti (2008) reviewed the effectiveness of the physical environment and crime on physical activity and highlighted the mediating impact of perceived safety and levels of neighbourhood crime. Where public transport was necessary and there were high levels of neighbourhood crime, traffic, or poorly maintained streets or lighting, individuals were less likely to engage. Associations between the perceived environment and transport were also reported in two studies (Gay, Saunders, & Dowda, 2011; Gothe & Kendall, 2016). Furthermore, costs of the activity itself (Withall, Jago, & Fox, 2011), distance, and travel problems (Ackerman, Buchbinder, & Osborne, 2013) were all also cited as key barriers to attendance.

#### 3.5.2 Engagement statement 2: If the transit to first session is supported THEN individuals may be more likely to attend

The second prioritised statement relating to Engagement related to the measures taken to mitigate the issues above and the practical support given to participants to help them feel informed, confident and able to attend the first session. Thirty‐eight included studies^4^ provided information that helped us understand what contributed to this transition.

Support to social prescribing activities was staged in terms of intensity and presented here in ascending order. First, patients could be assisted in their transition between referral and first session using introductory sheets which described what was proposed, the process, and included contact details: ‘Pre‐printed prescriptions reinforced [the referral] to patients…’ (Ackermann, Deyo, & LoGerfo, 2005). Introductory sheets could also be used as a facilitator to bring in a social prescribing link worker: ‘GPs…provide information and share relevant information with a…link worker’ (Bragg & Leck, 2017).

The second, and a slightly more connected approach, was a phone call post referral to assist with the transition and keep in contact with the patient. Often the link worker waited a few days and followed up each referral with a call which was thought to:

…enhance patient behavioural change after a community referral is made (Ackermann et al., 2005)

Increasing connectedness was the aim of the ‘buddy system’, whereby a link worker provided face‐to‐face support between referral and the first session and was thought to increase the likelihood of attendance. This contact varied in intensity and timescale, from a single contact: *If the patient chooses to engage…then this is followed by a more in‐depth guided conversation* (Bragg & Leck, 2017), through to much more intensive and multiple‐visit approaches where *a referred patient can have up to six sessions with the link worker*’ (Bragg & Leck, 2017) and:

the level and extent of…involvement…can differ greatly – from one‐off…to link workers accompanying…to the activity’ (Bragg & Leck, 2017)

Underpinning the above was a belief in the importance of networks to facilitate and increase the likelihood of a successful social prescription, with the assumption that the converse would be true:

patients who are simply given information about an opportunity will not necessarily take it up without some hand‐holding’ (Brandling & House, 2009)

Thus, ‘having someone to encourage or support’ (ERS Research & Consultancy, 2013) was considered central to successful referrals.

In our targeted searches, very few studies described the supported transit to the first session of a health activity. The first study examined the characteristics of a telephone follow‐up versus a group‐delivered diabetes prevention programme (Lim et al., 2017). Engagement was higher with the telephone follow‐up, with calls reported as an important motivator. Second was a review of interventions to improve Engagement with child mental health programmes; most effective were intensive link worker‐based models tackling practical or psychological barriers (Ingoldsby, 2010). This was echoed in Prado, Pantin, Schwartz, Lupei, and Szapocznik (2006), who described that the initial contact between facilitator and family was the strongest predictor of Engagement in an HIV prevention programme (Prado et al., 2006).

Perrino, Coatsworth, Briones, Pantin, and Szapocznik (2001) argued that it is important for any Engagement activity to occur prior to beginning a programme, and Williams and Sultan (1999) noted that these interactions should be culturally relevant to maximise assurance and encouragement.

### 3.6 Adherence (ongoing attendance)

#### 3.6.1 Adherence Statement 1: IF the leaders are skilled THEN the patient is more likely to maintain Adherence

Fifteen studies^5^ provided information relating to this theme, and from these sources we identified two themes contributing to our understanding of how the conditions of the statement are met.

First, the role and qualities of the leader of a social prescribing activity was central in maintaining Adherence:

The impact of the facilitator appears to influence directly the attendance of the patients; Diane: 'the numbers have kept up because she's so good, it's to her credit' (Mills, Crone, James, & Johnston, 2012)

Positive experiences of and relationships with activity leaders were thought to be associated with Adherence:

…[things] that would make them return to the gym included suitable qualified staff with more empathy with older people. (Martin & Woolf‐May, 1999)

Specifically, where a trusting relationship was developed the leader could help overcome barriers:

I think the participants were suspicious…at the beginning…some of them came because…they trust him, they know him. (Baker & Irving, 2016)

Mills et al. (2012) also reported that that ‘qualified staff with knowledge of medical conditions with appropriate exercise equipment and support’ reassured older participants and:

This safe environment is also reassuring to patients; Lydia: 'I like someone there to be watching what I am doing’ (Mills et al., 2012)

The second theme was the ways in which social prescribing activity leaders might maximise confidence among participants, with non‐judgemental concern, compassion, personal attention and advice being important, particularly where the perception of safety was a contributory factor:

…walking leaders described various methods they used to support participants including: providing constant encouragement; a friendly and positive attitude; empathising and engaging with participants, encouraging participants to mix… (Stirrat, 2014)

The use of cognitive‐behavioural, motivational and ‘persuasive’ techniques by activity leaders was linked to Adherence, and participants’ relationship with the leader also acted as a motivating factor:

Participants had found it particularly “helpful” and motivating that walking leaders did not appear to be “just going through the motions” but rather seemed “very enthusiastic” about their role: “they would make a point of talking to you and encouraging you… just showing an interest rather than just performing a function…they do seem genuinely interested in encouraging people” (Male Referred Participant). The fact walking leaders were volunteers had also acted as a motivator to attendance as participants felt they would have been “letting them down” by not turning up each week (Stirrat, 2014)

Studies largely did not follow‐up non‐completers, however, where reported unsupportive leadership was cited as a factor:

Non‐finishers were all asked 'what would make them come back to the gym' and there were some positive responses about returning to the gym. Factors that would make them return to the gym included suitably qualified staff with more empathy with older people (Martin & Woolf‐May, 1999)

The activity leader has a responsibility to encourage participants to continue to engage, and a lack of motivational skills could lead to individuals disengaging.

Evidence in our targeted searches also suggested that the real or perceived skill of the activity leader were instrumental in ongoing Adherence, these include: psychological support (Estabrooks et al., 2004; Izumi et al., 2015), motivational capacity (Caperchione, Mummery, & Duncan, 2011), trust (Estabrooks et al., 2004; Izumi et al., 2015), and promoting a positive environment (Estabrooks et al., 2004; Izumi et al., 2015).

#### 3.6.2 Adherence statement 2: If there is a change in patient's condition THEN the patient is more or less likely to maintain adherence

We coded data from 19 studies^6^ which provided information relating to this statement, and from these sources we identified two themes that contribute to our understanding of how the conditions of the statement are met.

One the key factors in ongoing Adherence was the perception that the social prescription resulted in change:

Another person who had been ‘prescribed’ an exercise and weight loss regime, was very clear about exactly what was motivating him. “Results! My cholesterol is right down, so I no longer need pills for that. And my blood sugar was extremely high when I was diagnosed, but it isn't now (ERS Research & Consultancy, 2013)

Besides physiological benefits participants reported that short‐term benefits like improved sleep, mood or simply enjoying the activity were common:

[Michael (63) said] ‘If I do not have physical activity I have difficulties in sleeping, but if I have physical activity sleeping is better.’ [Bennett (74) stressed that he gets up in the morning] ‘more easily and with a better mood (Stathi, McKenna, & Fox, 2004)

However, perceived lack of change in health status resulted in participants questioning the suitability of the activity:

…the main reason for drop‐out was disappointment at the lack of individual success. All these seven patients put on weight; for them the result was a failure in relation to their main motives for participation. Compared to the adherent group, these patients had no episodes of weight loss at all that they could relate to the experience of increased physical activity. (Jones, Harris, Waller, & Coggins, 2005)

The second theme related to expectations of what could be achieved through the social prescription; potentially those with higher or unrealistic expectations were least likely to maintain Adherence:

[he was] …concerned about his ability to achieve the kind of results he needed (ERS Research & Consultancy, 2013)

Jones et al. (2005) reported that those failing to complete the offered sessions had greater expectations of change than completers:

…false hopes may exist amongst participants…highly unrealistic expectations…[and] suggested that those who had greater expectations of change over a 10‐week prescription were least likely to finish (Jones et al., 2005)

Importantly, external factors such as difficulty in making life changes or the expectations of others also moderated Adherence:

Our youngest daughter was saying ‘Mummy’s going to the gym, she'll never keep it up. ’Anyhow, mother did and mother felt considerably better for it. (Joan, age category 55‐64; (Jones et al., 2005))

Our targeted searches for evidence for this statement again located very few studies. Burridge et al. (2016), in their qualitative exploration of diabetes self‐care, supported our finding that shifts in health status contributed to Adherence to (often burdensome) programmes of self‐care.

### 3.7 Summary

**Figure 3:**
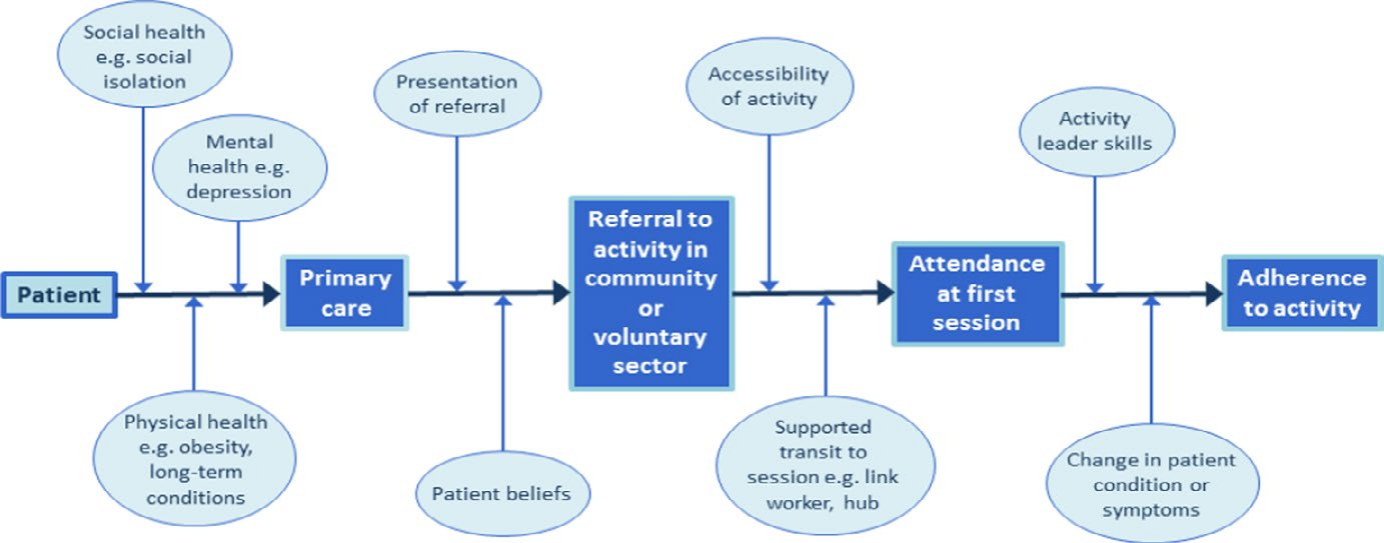
Social prescribing pathway [Colour figure can be viewed at http://www.wileyonlinelibrary.com/]

Figure 3 below illustrates the social prescribing pathway along which individuals are introduced to and then navigate services and along which the six prioritised theories sit (the right‐hand six ovals):

Patient motivation, self‐efficacy and a belief in the relevance of the activity, which all impacted on the acceptance and uptake of activities are areas not always considered in GP consultations (Alexander et al., 2011). The *way* in which an activity was presented also affected patient action. For example, someone visiting their GP and expecting a physical prescription may be more likely to accept and attend if they received written information about it (Dempster et al., 2015; Himmel et al., 1997).

More broadly, receptiveness to referral was also influenced by cultural characteristics (Garrett et al., 2012; Hudson et al., 2016), potentially requiring differential presentation. Recognition of patient needs alongside GP preference is therefore key to effective referral. After acceptance, the next step in the process is for the patient to attend the prescribed activity (Engagement). Evidence located pointed to cost, transport, venue and time impacting on the likelihood of attendance. Reminder phone calls, written information, introductory sessions, or attendance with a ‘buddy’ have been used to maximise attendance for different groups.

Following attendance at the first session, evidence suggests that trained staff exhibiting good leadership, an activity fostering interpersonal relationships and trust, supportive environments, as well as an individuals’ perceived change in condition, and an absence of negative effects determine continued attendance. Throughout all three stages, the social prescribing process can be modified to take these factors into account.

## 4 DISCUSSION

Social prescribing is receiving increasing government backing in the UK, however, the evidence base for what works, for whom and in what circumstances lags behind the enthusiasm for implementation.

This review of 109 studies produced theory relating to the ways in which the referral process might be implemented for different groups across our three organising principles: Enrolment, Engagement and Adherence, and provides explanatory detail for six key areas. These areas were prioritised by an Expert Advisory Group, with others left for future analyses. Studies indicate that patients are more likely to *enrol* if they believe the social prescription will be of benefit, if the referral is presented in an acceptable way that matches their needs and expectations, with concerns elicited and addressed appropriately by the referrer. Patients are more likely to *engage* if their chosen activity is accessible and transit to the first session supported. *Adherence* to programmes is impacted through skilled and knowledgeable activity leadership or through changes in conditions or symptoms. Included studies were often lacking in theoretical descriptions, however, well‐established behaviour change theories can help us make sense of these findings. Where patients’ belief of benefit impacts on enrolment, for example, Bandura (1978, 2008) model of self‐efficacy is relevant, in which an individual's confidence in their ability to exert control and produce desired effects is the driving force behind action. Similarly, Leventhal's Common Sense model of illness (Diefenbach & Leventhal, 1996), which outlines the processes by which individuals form representations of health threats and their responses in relation to them, goes some way to explaining differences in perceived effectiveness of the prescribed activity.

However, the evidence base is not sufficiently developed to make general inferences about effectiveness of particular approaches. Indeed, what constitutes ‘effectiveness’ for such complex pathways (see below) is difficult to define. There is much in the policy literature seeking to link social prescribing with reduced health service use, and it is possible this is the case, however, there is evidence in this review that the converse may also be true in some instances; with previously unengaged individuals seeking a referral through their GP, and the offer of much broader interventions addressing previously unmet need.

The evidence base is also lacking detail around contextual contingency; for example, data relating to the impact of psychological characteristics, condition or type of activity, is crucial to our understanding, however, currently this is absent.

Given the recent promise that all Primary Care Networks are to have NHSE‐funded link worker roles (NHS England, 2018), it is important to consider their impact on the social prescription process. We assert that link workers are necessary, they have the potential to contribute to multiple elements of successful uptake, but not sufficient to the smooth running of the pathway. Our analysis indicates that well‐trained and knowledgeable link workers are beneficial for accessing, developing knowledge of activities and assisting transitions between services. However, social prescribing is not a single intervention but a pathway with many interacting elements. It is also a series of relationships, between referrer and patient, patient and link worker, link worker and activity and patient and activity, all of which need to function to meet patient need. These combine and interact with local contexts and the patient's social, mental and physical health to affect the referral's success.

Despite the lack of high‐level evidence there seems sufficient explanatory detail to suggest that social prescriptions are more likely to be successful with these inputs, and specifically amongst those with complex needs.

### 4.1 Strengths and limitations

A major strength of this review is the focus on the *process* of social prescribing. Few studies have tried to understand *how* to get people from their GP to a social prescribing activity. We used realist approaches to surface mechanisms for the process, which allows our findings to be transferable across settings and activities.

Another strength is the breadth of evidence we include, we conducted extensive grey literature searches and contacted relevant organisations—many social prescribing programmes are not reported in academic papers and therefore would not have been located through only database searches (Cooper et al., 2018). The involvement of experts through our Expert Advisory Group helped to refine our searching and inclusion criteria with their insider knowledge of relevant terms and activities. This group was also central in developing and testing our programme theories and in prioritising the areas for us to focus on through more targeted searches. Whilst forming a core part of the realist synthesis approach, these targeted searches are themselves a strength of this review. The searches meant that these elements were all strengthened in terms of providing better explanations of programme theories, and to strengthen inferential explanations.

We accept, however, that time and resources constraints meant it was not possible to consider all of the statements in detail. The strength of the findings is also limited by the majority of the studies relating to exercise prescriptions, particularly those identified through database searches, with pockets of evidence relating to specific activity types (i.e. arts on prescription); for many areas little or no evidence was identified. Our ability to make nuanced inferences was also limited owing to the lack of detailed descriptions in studies.

### 4.2 Comparisons with existing literature

Although our study was unique in its focus only on the *process* of social prescribing, our findings are in line with Pilkington et al. (2017) scoping study, where the team found limited evidence owing to information not being published and activities not being labelled as ‘social prescribing’.

We restricted our criteria to referrals from primary care. However, Chatterjee et al. (2017) found that the referral pathway has broadened to include referrals from practice nurses, physiotherapists, as well as from health professionals outside of primary care. Although this may reduce GP burden (Chatterjee et al., 2017), it adds complexity in defining and identifying initiatives. Our review and recent literature are agreed that the link worker model and personalisation of the support, regardless of the original referral, is one of the more important features in effective social prescribing initiatives (Chatterjee et al., 2017; Moffatt, Steer, Lawson, Penn, & O'Brien, 2017; Pilkington et al., 2017).

### 4.3 Recommendations

The evidence presented here highlights important considerations in developing social prescribing practice. First, it is important for the social prescribing programme and activities to be responsive to the context, for instance if transport is needed to access the activity, it is necessary for that transport to be available and affordable for the referee. To encourage adherence professionals leading the activities must have appropriate interpersonal and leadership skills to create a trusting environment which fosters realistic expectations of change. The review findings supported the recent investment in providing a link worker role for each Primary Care Network, the inclusion of a link worker appeared to be vital. Matching the referral to patients on an individual basis according to patients’ needs, personality and cultural background is crucial and should continue to be supported.

Opportunities to return to the link worker after attending a service for further support is also recommended (Model 3+).

For all programmes, it is important to develop social prescribing in line with complex intervention and behaviour change approaches with a careful consideration of context and capacity. This is new ground and there is a pressing need for theory‐informed practice, not only because theory‐driven interventions are more likely to be effective (Denford et al., 2015) but also because successful implementation of social prescribing programmes involves behaviour change on the part of both practitioners and participants; there is an extensive literature relating to health behaviour change and it is important that planners draw on this when designing programmes. Linked to this, it is also important that there is high‐quality research developed alongside practice, and we have argued elsewhere that whilst this is difficult to do robustly in such a complex system there are some keys ways in which it might be achieved (using evidence to inform elements of the patient pathway, reporting contextual factors, and being realistic about what outcomes are relevant and useful; Husk, Elston, Gradinger, Callaghan, & Asthana, 2019).

## 5 CONCLUSIONS

We suggest that with the proliferation of social prescription programmes, the interest, investment and innovation be harnessed within a high‐quality concomitant research programme. This programme should, as well as assessing effectiveness on outcomes where possible, report the target populations, baseline characteristics, acceptability, reach and scope of services. The evidence examined here indicates the level of complexity necessary for robust implementation, and so services need to better understand what it is that patients need in terms of complex care. Signposting at the point of presentation for individuals with mental health needs, for example, is not likely to be sufficient. Conversely, even with deep understandings of those needs and robust links between health and provider services, social prescriptions are unlikely to be a panacea and effectiveness will be dependent on complex interactions and relationships between patient, context, resources and services.

## COMPETING INTERESTS

All of the authors declare they have no competing interests.

## AUTHOR CONTRIBUTORS

KH and RG led the review, KB and RL were the co‐reviewers. AB was the information specialist for the review. MP provided guidance in the support of realist methodology. RB, DB and SW conceived of the project and contributed to the development of the manuscript. RB and IL provided methodological and executive leadership support. All authors read and approved the final manuscript.

## ENDNOTES

1 (Ackermann et al., 2005; Adsett et al., 2013; Bragg & Leck 2017; Brandling & House, 2007, 2009; Brandling et al., 2011; Callaghan et al., 2016; Campbell et al., 2015; Din et al., 2015; Duda et al., 2014; Edmunds et al., 2007; ERS Research and Consultancy, 2013; Graham et al., 2005; Hammond et al., 1997; Harrison et al., 2005; Jolly et al., 2009; Jones et al., 2005; Mills, 2008; Moore et al., 2011; NHS England, 2016; Popay et al., 2007; Rahman et al., 2011; Roessler, 2011; Stirrat, 2014).

2 (Adsett et al., 2013; Baker & Irving 2016; Bragg & Leck 2017; Brandling & House 2009; Brandling et al., 2011; Bungay & Clift 2010; Callaghan et al., 2016; Din et al., 2015; Duda et al., 2014; ERS Research and Consultancy, 2013; Friedli et al., 2012; Gidlow et al., 2005; Graham et al., 2005; Jepson et al., 2010; Mills, 2008; Moore et al., 2011; Pavey, Anokye, et al., 2011; Popay et al., 2007; Roessler, 2011; Schmidt et al., 2008; Sealey, 2015; Stirrat, 2014; Walker & Thirlwall 2015; Wormald et al., 2006).

3 (Adsett et al., 2013; Bragg & Leck 2017; Brandling & House 2007; Brandling et al., 2011; Browne, 1997; Callaghan et al., 2016; Campbell et al., 2015; Crone et al., 2004; Din et al., 2015; Duda et al., 2014; Edwards et al., 2013; Elton et al., 2005; ERS Research and Consultancy, 2013; Fox, 1997; Gidlow et al., 2005; Gidlow et al., 2007; Hammond et al., 1997; Martin & Woolf‐May 1999; Maynard, 2012; Moore, Raisanen, Moore, Ud Din, & Murphy, 2013; Morton et al., 2008; Parsfield et al., 2015; Pavey, Taylor, et al., 2011; Schmidt et al., 2008; Stirrat, 2014; Tai et al., 1999; Tava & Nosa 2012; Vogelpoel & Jarrold 2014).

4 (Ackermann et al., 2005; Baker & Irving 2016; Bragg & Leck 2017; Brandling & House, 2007, 2009; Brandling et al., 2011; Brown et al., 2004; Browne, 1997; Callaghan et al., 2016; Campbell et al., 2015; Din et al., 2015; Dinan et al., 2006; Duda et al., 2014; Edmunds et al., 2007; Elton et al., 2005; ERS Research and Consultancy, 2013; Friedli, Vincent, & Woodhouse, 2007; Friedli et al., 2007; Hammond et al., 1997; Haynes, 2011; Jolly et al., 2009; Kimberlee, 2013, 2016; Kimberlee, Ward, Jones, & Powell, 2014; LVSC, 2016; Maynard, 2012; Mills et al., 2011; Moore et al., 2011; NHS England, 2016; Pavey, Taylor, et al., 2011; Rahman et al., 2011; Sealey, 2015; Stirrat, 2014; Tava & Nosa 2012; Vogelpoel & Jarrold 2014; Walker & Thirlwall 2015; Wood & White 2011; Wormald et al., 2006).

5 (Baker & Irving 2016; Crone et al., 2005; ERS Research and Consultancy, 2013; Martin & Woolf‐May 1999; Mills, 2008; Mills et al., 2012; Moore et al., 2011; Roessler, 2011; Stathi et al., 2004; Stickley & Hui 2012; Stirrat, 2014; Tava & Nosa 2012; Williams et al., 2007; Wormald & Ingle 2004; Wormald et al., 2006).

6 (Bragg & Leck 2017; Brandling & House 2007; Brandling et al., 2011; Callaghan et al., 2016; ERS Research and Consultancy, 2013; Haynes, 2011; Jones et al., 2005; McNair, 2006; Mills, 2008; Moore et al., 2011; Moore et al., 2013; Pavey, Taylor, et al., 2011; Roessler, 2011; Stathi et al., 2004; Stickley & Hui 2012; Stirrat, 2014; Tava & Nosa 2012; Vogelpoel & Jarrold 2014; Wormald & Ingle 2004).
