# Supplementary material for: What approaches to social prescribing work, for whom, and in what circumstances? A realist review
Source: Health Soc Care Community. 2019 Sep 9;28(2):309–24. doi: 10.1111/hsc.12839 (PMC7027770; doi:10.1111/hsc.12839)
Supplement: Supplementary file 1 [file HSC-28-309-s001.pdf]

Appendices

Appendix 1 - Definition boxes

**If-then statements**	A common way of expressing theories about how a programme works in a number of linked statements taking the form of: 'if X, then Y'.
**Rigour and relevance**	Studies were assessed using judgements about whether a source was conceptually-rich (with well-grounded and clearly described theories and concepts, thick (a rich-description of a programme, but without explicit reference to theory underpinning it), or thin (weaker description of a programme, where discerning theory would be problematic).
**Community based activities**	Which can include exercise, arts, and nature programmes. There was literature around 'exercise prescription' in Australia which related to simply directing individuals to increasing exercise and where there was no referral. We excluded these studies.
**Articles**	As described in Livoreil et al. Environ Evid (2017) 6:23 (https://environmentalevidencejournal.biomedcentral.com/articles/10.1186/s13750-017-0099-6): "In this paper we use "article" to refer to any written document including scientific papers, abstracts, reports, book chapters, other publications, thesis, or internet pages, etc. Articles may contain more than one study (described observation or experience including methods and results) or the same study may be reported in more than one article. In a systematic review or map, the unit of analysis (especially when conducting a meta-analysis) is the study." Studies appearing in more than one article were only included once.

Appendix 2 – Searches (a)

The following databases were searched in November 2015 for stage (a):

- Medline & Medline in process (Ovid)
- Embase (Ovid)
- CINAHL (EBSCO)
- WoS (Thompson)
- SportsDiscuss (ProQuest)
- Greenfile (EBSCO)
- Scopus (Elsevier)
- ASSIA (ProQuest)

We also searched the following websites,

We also searched the first 100 hits of standard Google queries, using the following terms:

We assessed rigour and relevance to the question using a classification tool (Mark REF 2012), which classifies studies as 'conceptually rich' (with well-grounded and clearly described theories and concepts), 'descriptively thick' (rich descriptions of a programme but without explicit reference to the

theory underpinning it), or 'descriptively thin' (weaker descriptions of a programme, where discerning theory would be problematic).

Appendix 3 – Organisations and individuals contacted for grey searching

We contacted each member of our EAG and followed leads they provided to online, telephone or individual contacts. We also used our own contacts as academic authors to do the same, as well as free searching the below organisations:

**Organisation**	**Website**	
Rushey time bank	http://www.rgtb.org.uk/	
Wellbeing enterprises	http://www.wellbeingenterprises.org.uk/	
Bromley by Bow centre	http://www.bbbc.org.uk/	
Connect for health	https://connectforhealthleeds.org.uk/learn-more	
Growing well. Cumbria	https://growingwell.co.uk/referrers/	
Rotherham Social Prescribing Service	http://www.varotherham.org.uk/social-prescribing-service/	
natural health service merseyside	http://www.naturalhealthservice.org.uk/	
Ways to Wellness	http://waystowellness.org.uk/	
Wellspring Healthy Living Centre, Bristol	http://www.wellspringhlc.org/	
Woodland Wellbeing Project	http://forestofavontrust.org/2016/06/woodland-wellbeing/	
West Leeds Patient Empowerment Project	http://barca-leeds.org/health-and-wellbeing/west-leeds-pep-patie	
Natural Health Service for Weymouth and Portl	https://www.dorsetforyou.gov.uk/natural-health-service	
**search number (corresponds to folders)**	**terms**	**extent of search (pages/hits**
Google search 1	social prescription evaluation	first 10 pages (100 hits)
Google search 2	community prescription evaluation	first 10 pages (100 hits)

Appendix 4 – Supplementary Search results (b) and Prioritised element CMO detail

We searched MEDLINE for each of the three organising principles and identified the following:

**Domain**	**Statement**	**Hits (N)**	**Included (N)**
Enrolment	IF the patient believes…	371	11
	IF the referral is presented…	87	7
Engagement	IF the activity is accessible…	1004	8
	IF the tranist to the first session…	40	1
Adherence	IF the leader(s) are skilled…	282	6
	IF there is a significant change…	104	1

Search 1. Enrolment

Statement 1

Self efficacy.ti	Primary care
Self determination theor*	GP
SDT	General practitioner*
health belief model*	Family physician*

HBM	
Theor* of Reasoned Action*	
TRA	MeSH terms for above
Theor* of Planned Behaviour*/behavior*	
TPB	
patient motivation*	

Statement 2

Candidacy	Primary care
Referral mechanism*	GP
(Treatment option) adj2 accept*	General practitioner*
Referral adj2 presentation	Family physician*
Need* adj2 satisfaction*	
	MeSH terms for above

Search 2. Engagement

Statement 1

Barrier*		
Facilitator*	Group adj2 (activit* or program* or intervent*)	
Accessibil*		
Affordab*		
Transport*		
Environment		
neighbourhood		

Statement 2

informed	Theor*		
Information			
Hand holding			
Buddy			
Introduction			
Link worker			
Reminder			
taster			

Tried the above first but was getting 1,000s hits using ti or tw field codes so decided on the following search:
(Group adj2 (activit* or program* or intervent*)) AND theor*.tw AND engagement.tw

We located so few studies in MEDLINE, we expanded our search to Google Scholar with the same terms and identified four others.

Search 3. Adherence

Statement 1

Leadership	Group (activit* or program* or intervention*)

Leader	
Health communication	
Charisma	
Social intelligence	
Trust	
Perceptions of safety	

Statement 2
Group (activit* or program* or intervention*) AND adherence.tw AND theor*.tw
All of these results are a subset of the search for statement 1

Appendix 5 – Overarching Programme Theory

This is a separate document, please see supplementary material.

Appendix 6 – Tabulations of quotes from prioritised statements

Enrolment Statement 1: IF the patient believes the social prescribing will do them good THEN they may be receptive and enrol.

If-then statement	Theme	Quote	Reference
If the patient believes the social prescribing will do them good then they may be receptive and enrol.	Patient expectations of the consultations and solution seeking behaviour in the consultation	*"I was pleased. I was struggling to control my diabetes and I thought this would help. I was feeling really down and [my GP] suggested this so I could lose weight and do something for me."*	ERS Research and Consultancy 2013
If the patient believes the social prescribing will do them good then they may be receptive and enrol.	Patient belief that they have a condition the social prescribing will address	*"As with most people, exercise isn't really my thing so I wasn't that keen, but I decided to give it a go. I had just been diagnosed with type 2 diabetes and the practice nurse at West Road referred me straight away so I could start to deal with it with diet and exercise". "It was my idea . . . I wanted to do something that would help me get better and control the pain in my joints. The GP agreed and referred me."*	ERS Research and Consultancy 2013
		"The Health Belief Model (HBM) is one of the most established social cognition models. The HBM uses two aspects of individuals' representations of health behaviour in response to threat or illness. These are the perception of illness threat and evaluation of behaviours to counteract this threat"	Mills 2008
		"A second major theme in these accounts was the persistence with which people sought solutions to their problems, often despite formidable psychological, social and/or material obstacles. Whilst in most cases these actions of self-agency had not resulted in sustainable solutions at the time of interview, they do highlight the resourcefulness displayed by these respondents amidst great adversity. In some accounts, although often tentative, people felt that the steps they had taken had the potential to transform their lives"	Popay 2007
If the patient believes the social prescribing will do them good then they may be receptive and enrol.	The belief that the social prescribing provider is reliable	*"For participants, the most common barriers were concerns regarding staff training or appropriate facilities to manage complex patients"*	Adsett 2013

Enrolment Statement 2: IF the referral is presented in an acceptable way and matches patient needs and expectations THEN they may be receptive and enrol.

If-then statement	Theme	Quote	Reference
If the referral is presented in an acceptable way and matches patient needs and expectations then they may be receptive and enrol.	The specifics of the activity on offer	*"Professionals reported that the idea … provoked anxiety for many patients, with initial anxieties perceived as stemming from worries about having confidence undermined by the presence of fitter exercisers, fears about assimilating into an unfamiliar social environment or fears of being expected to do exercises they weren't able to do. They are just worried about what people will think of them, they think the people there, everyone there is going to be fit, in their lycra and looking really smart but so that's the main thing, they just don't know, it's the fear of the unknown, they don't know what we are going to do with them"*	Moore 2011
		"They say yes, it's quite daunting coming into the leisure centre for the first time, they're not too sure what they are going to be doing ...so we are trying to design a leaflet now which we are going to put out with the card itself saying exactly what they are required to do"	Moore 2011
		"Initial consultations were often cited as an opportunity to reassure patients that professionals would serve as a familiar point of contact, as well as offering assurance that patients would not be expected to do anything that they were not confident about doing or which made them uncomfortable. Highlighting at this stage that the patient would be surrounded by others in the same position was seen as playing a substantial role in assuaging anxieties"	Moore 2011
		"Many of the participants had not been given an information leaflet upon referral, so had no idea what to expect"	Wormald 2006
If the referral is presented in an acceptable way and matches patient needs and expectations then they may be receptive and enrol.	The referral process itself	*"…GP participant recognised that a social prescription may be accepted by a patient just because it has the credibility of being the doctor's suggestion."*	Brandling and House, 2011
		"Many Dutch experienced the advice as being 'just a recommendation', which meatn it was not experienced as a deciding factor…many migrant participants, however, experienced the GP as someone 'who knows better' and participated in the intervention because they were told to do so."	Schmidt 2008
		"So I had to change my consultation style to enable me to open up a discussion about social prescribing and if the patient was interested."	Frieldli 2012
If the referral is presented in an acceptable way and matches patient needs and expectations then they may be receptive and enrol.	The format and delivery of the referral	*"…referral forms were provided to all…and completed on behalf of interested participants."*	Adsett 2013
		"If an individual was considered to meet the referral criteria, the project was discussed with them."	Baker 2016
		"Referrers should be made aware that the interactions during referral have a strong contributing effect on whether patients engage with the service offered."	Brandling and House 2007
If the referral is presented in an acceptable way and matches patient needs and expectations then they may be receptive and enrol.	What the patient presented with in terms of symptoms	*"So we talked through my situation and she wrote down the topics that I particularly wanted to be helped with. And I was really pleased to be able to have this attention, because sometimes you just don't know who to go to to ask these things, you know…And they weren't particularly things that GPs would necessarily…you know, that you would necessarily, sort of, bother them with, if you like…..(Participant)"*	Callaghan 2016
		"Commonly patients with pre-existing illnesses, e.g. high blood pressure or heart disease, are fearful that engaging in physical activity will exacerbate their condition; similarly older individuals are often fearful of getting injured"	Stirrat 2014

Engagement Statement 1: IF the activity is accessible to the patient THEN they are more likely to attend.

If-then statement	Theme	Quote	Reference
If the activity is accessible to the patient then they are more likely to attend.	Cost	*"…such as cost…were seen as advantages and disadvantages of the community-based program, depending on individual circumstances."*	Adsett 2013
If the activity is accessible to the patient then they are more likely to attend.	Proximity and safety	*"the neighbourhood setting was given as a reason…participants do not feel safe in their neighbourhoods…and this was a reason to stay home."*	Schmidt 2008
If the activity is accessible to the patient then they are more likely to attend.	Time of day	*"[attendance being impacted by] seasonal changes in lighting."*	Stirrat 2014
If the activity is accessible to the patient then they are more likely to attend.	Safety and allocation of transport	*"…[the most] valuable form of support…was transport to appointments."*	Callaghan 2016
		"…the only significant correlates of uptake…were car ownership and deprivation."	Campbell 2015
		"…many in deprived are practices, especially, were concerned that patients might revert to their previous routine once the subsidised period was over."	Din 2015

Engagement Statement 2: IF the transit to first session is supported THEN individuals may be more likely to attend.

If-then statement	Theme	Quote	Reference
If the transit to the first session is supported then individuals may be more likely to attend	Introductory sheets	*"Pre-printed prescriptions reinforced [the offer] to patients"*	Ackermann 2005
		"GPs…provide infromation and share relevant information with a…link worker"	Bragg 2017
If the transit to the first session is supported then individuals may be more likely to attend	Phone contact	*"…follow up phone calls…to enhance patient behavioural change…"*	Ackermann 2005
If the transit to the first session is supported then individuals may be more likely to attend	A 'buddy' system of support varying in intensity	*"If the patient chooses to engage…then this is followed by a more in-depth guided conversation"*	Bragg 2017
		"a referred patient can have up to six sessions with the link worker"	Bragg 2017
		"the level and extent of…involvement…can differ greatly - from one-off…to link workers accompanying…to the activity"	Bragg 2017
If the transit to the first session is supported then individuals may be more likely to attend	Networks	*"[assumed that] patients who are simply given information about an opportunity will not necessarily take it up without some hand-holding"*	Brandling 2009
		"[important] having someone to encourage or support"	ERS 2013

Adherence Statement 1: IF the leaders are skilled THEN the patient is more likely to maintain Adherence.

If-then statement	Theme	Quote	Reference
If the leaders are skilled then the patient is more likely to maintain adherence	Perceived skill of the leader	*"The impact of the facilitator appears to influence directly the attendance of the patients; Diane: 'the numbers have kept up because she's so good, it's to her credit'"*	Mills 2012
		"…[things] that would make them return to the gym included suitable qualified staff with more empathy with older people."	Martin 1999
		"I think the participants were suspicious of me at the beginning…some of them came because…they trust him, they know him."	Baker 2016
		'qualified staff with knowledge of medical conditions with appropriate exercise equipment and support'	Mills 2012
		"This safe environment is also reassuring to patients; Lydia: 'I like someone there to be watching what I am doing'"	Mills 2012
If the leaders are skilled then the patient is more likely to maintain adherence	The ways in which activity leaders might maximise confidence among participants	*"…walking leaders described various methods they used to support participants including: providing constant encouragement; a friendly and positive attitude; empathising and engaging with participants, encouraging participants to mix…"*	Stirrat 2014
		"Participants had found it particularly "helpful" and motivating that walking leaders did not appear to be "just going through the motions" but rather seemed "very enthusiastic" about their role: "they would make a point of talking to you and encouraging you… just showing an interest rather than just performing a function…they do seem genuinely interested in encouraging people" (Male Referred Participant). The fact walking leaders were volunteers had also acted as a motivator to attendance as participants felt they would have been "letting them down" by not turning up each week"	Stirrat 2014
		"Non-finishers were all asked `what would make them come back to the gym' and there were some positive responses about returning to the gym. Factors that would make them return to the gym included suitably qualified staff with more empathy with older people"	Martin 1999

Adherence Statement 2: IF there is a change in patient's condition THEN the patient is more or less likely to maintain Adherence.

If-then statement	Theme	Quote	Reference
If there is a change in the patient's condition then the patient is more or less likely to maintain adherence	Changes in symptoms or condition	*"Another person who had been 'prescribed' an exercise and weight loss regime, was very clear about exactly what was motivating him. "Results! My cholesterol is right down, so I no longer need pills for that. And my blood sugar was extremely high when I was diagnosed, but it isn't now"*	ERS 2013
		"…the main reason for drop-out was disappointment at the lack of individual success. All these seven patients put on weight; for them the result was a failure in relation to their main motives for participation. Compared to the adherent group, these patients had no episodes of weight loss at all that they could relate to the experience of increased physical activity".	Jones 2015
		"[Michael (63) said] 'If I do not have physical activity I have difficulties in sleeping, but if I have physical activity sleeping is better.' [Bennett (74) stressed that he gets up in the morning] 'more easily and with a better mood"	Stathi 2004
If there is a change in the patient's condition then the patient is more or less likely to maintain adherence	Changes in expectations	*"[he was] …concerned about his ability to achieve the kind of results he needed"*	ERS 2013
		"…false hopes may exist amongst participants…highly unrealistic expectations…[and] suggested that those who had greater expectations of change over a 10-week prescription were least likely to finish"	Jones 2005
		"Our youngest daughter was saying 'Mummy's going to the gym, she'll never keep it up. 'Anyhow, mother did and mother felt considerably better for it."	Jones 2005
