# Supplementary material for: What approaches to social prescribing work, for whom, and in what circumstances? A realist review
Source: Health Soc Care Community. 2019 Sep 9;28(2):309–24. doi: 10.1111/hsc.12839 (PMC7027770; doi:10.1111/hsc.12839)
Supplement: Supplementary file 2 [file HSC-28-309-s002.pdf]

Social Scripts – Programme Theory Development

We derived the below 'if-then' statements from our first round of searches: databases were trawled according to the search strategy listed and hits double screened and f/t included which met our criteria. These were refined through the conceptually rich includes from grey searches. All were read by the team and salient sections were read in full day sessions with all the review team and listed under 'patient, GP[1], process and activity' headers. We then refined these data to fit under our three primary outcomes of 'enrolment, engagement and adherence' but still relating to the chronological themes.

We conceptualise (where 'link' might be a person, database or app):

'1': Signposting (GP only)

'2': Light (GP – activity)

'3': Moderate (GP-link-activity / GP-link-volunteer-activity)

'3+': Holistic (GP-link (-volunteer) – activity, where link if flexible and iterative).

---

1. Enrolment
    a. Patient

IF the patient is unhappy with their current care OR alternative options THEN then they may be receptive to social prescribing.

IF the patient believes (expectations/they have condition it will address/GP consultation theory/attainment/provider is reliable) the SP will do them good THEN they may be receptive.

IF the patient knows about the activity (taster days/£ for normal attendance so referral free/peer recommendation) and whether it is appropriate/effective/desirable THEN they may request referral.

IF the patient believes they can access (link worker/physical location/online) the process THEN they may be receptive.

[1] NB: We use GP as shorthand for ease, we mean primary care practitioner.

IF the patient believes they can access (transport/time of day/£/childcare or other dependent/psychological/costs, i.e. benefit reduction) the activity THEN they may be receptive.

IF the patient is motivated (imminent fear of consequence, desperation with situation) THEN they may be receptive.

IF the referral is presented (specifics of activity) in an acceptable (referral process/how they respond/order vs questions) way and matches patient needs and expectations (what they think is wrong with them) THEN they may be receptive.

---

1. Enrolment
    b. GP

CONTEXTS [10-minute consultation (opportunity costs) / who doing referral under what circumstances / under RCT or study conditions / organisational support (social impact bonds, CCG, 5yr forward, GP forward view)]

↕

IF the GP knows (database/contacts/colleague/professional network/coordinator/study protocol/provider contact/time to learn) about the activities THEN they may be more likely to provide referral.

IF GPs experience (visit or trial/reputation/previous experience (through feedback, short concluding summaries)) the intervention THEN they are more likely to (a) refer, and (b) act as advocates in the practice.

IF the GP has the resources (database/time/knowledge) to refer THEN they may be more likely to refer.

IF the opportunities match practice-level delivery priorities (belief vs reality of frequent attenders) THEN the GP more likely to refer.

IF the GPs personal belief (knowledge/experience) is that the intervention or process is effective THEN they are more likely to refer.

Sub: IF the GP trusts (delivery/capacity/reliability/'mechanism'/accredited) the intervention (feedback/experience) THEN they are more likely to refer.

IF GPs are persuaded (questionnaire/GP assessment/screening/capacity of activity or prioritisation) of a patients' readiness (motivation/health condition) to attend THEN they are more likely to refer.

IF the GP is comfortable in the role (duty or ability to address 'non-medical' issues/trying SP) of referrer to SP THEN they are more likely to refer.

Sub: IF the GP is comfortable with the legality of liability for the intervention (feedback/experience) THEN they are more likely to feel comfortable in the role.

IF the GP has exhausted other viable options (something rather than nothing) THEN they are more likely to refer.

IF the GP feels this complements the existing treatment regime (in dialogue with patient) THEN they are more likely to refer.

---

1. Enrolment
   c. Process

CONTEXT [continuum from: information given - handholding]

IF the options are collated (facilitator which can be external or peer and professional/database/help hub/booklet) THEN GPs/patients are more informed.

Sub statements of the above:

- IF the collation is a booklet THEN it is easy to show the patient the options, but it's less formal and harder to update.
- IF the collation is a database THEN GPs can search, it is updateable but more expensive, someone needs to build and maintain it.
- IF the collation if managed by a facilitator THEN GPs more likely to know about interventions and easier for GP. But expensive and another potential exit point for the patient (adherence).
- IF the collation is facilitated by training or taster days THEN GPs are more likely to know about sessions and have confidence in them.
  - IF this continuum is facilitated by a volunteer link worker THEN availability (+/-) and sustainability (+/-) is impacted.

IF the referral includes a formal prescription document THEN this may influence the patients' beliefs and perceptions about the referral.

IF the referral process is flexible (i.e. '3+') THEN the GP has more options to be more responsive. OR

IF the GP has a protocol to follow THEN the GP may feel more confident to refer.

IF the link worker and patient have multiple contact sessions pre-activity THEN patient more likely to identify suitable activity.

---

1. Enrolment
    d. Activity

IF the activity is described/marketed in an appealing and appropriate (i.e. suspicion of marketing) (communication/reliability/see ‘APPROPRIATE LEAD’ below) way THEN the patient is more likely to attend/enrol.

IF the activity is demonstrated/marketed as cost-effective THEN the GP practice is more likely to adopt as an option.

IF there is a choice of activity THEN the patient is more likely to find one suitable/interesting.

IF there is a tailoring of activity THEN the patient is more likely to find one suitable/interesting.

IF there is robust data monitoring and feedback to the GP THEN they are more likely to refer.

IF the patient experiences a delay for entry THEN patients are less likely to attend and engage.

IF the activity is led by an appropriate (trained/reliable/volunteer status/communicated) leader THEN the patient and GP are more likely to be confident.

IF the lines of communication are open and smooth (i.e. admin/clerical hurdles) THEN the GP will (a) know the providers and (b) be responsive to referral.

---

2. Engagement
    a. Activity

IF the activity <u>is</u> accessible (cost/local/timing/safe/transport) to the patient THEN they are more likely to attend.

IF the activity provides taster sessions THEN the GP and patient are more likely to have knowledge of activities. (+therefore patient attend? link to enrolment)

---

2. Engagement
    b. Patient

IF the patient maintains their motivation (timescale / discussion of other treatments / reflection) THEN they may be more likely to engage.

---

2. Engagement
    c. Process

IF the transit to first session is supported (phone call/buddy/intro sheets/hub/network) THEN they may be more likely to attend.

---

3. Adherence
    a. Process

IF the <u>facilitating</u> (skills/facilitator/GP/buddy) mechanism maintains <u>contact</u> (drop outs) THEN the patient may maintain adherence.

IF there is an <u>end goal/target</u> (become buddy/reward for completion/graduate/reduce symptoms) THEN this may help the patient maintain adherence.

IF the process is iterative and flexible to learn from dropouts and completers THEN it may help encourage adherence amongst other patients.

---

3. Adherence
    b. Activity

IF the leader is skilled (communication/activity/health condition) THEN patient is more likely to maintain adherence.

IF the activity continues to be accessible THEN the patient will maintain adherence.

IF the first impression of the activity matches THEN the patient may be more likely to maintain adherence.

IF the activity continues to meet expectation (enjoyable/appropriate/fit to daily life) THEN the patient may be more likely to maintain adherence.

IF the activity is group based (religion/appropriate/coherence/size/ethnicity/gender/ability) THEN it may increase adherence for some through social support (and vice versa).

IF the activity duration, frequency and regularity suit patient THEN they are more likely to adhere.

---

3. Adherence
    c. GP

IF the GP receives robust and appropriately fed back data for individual patients (formal from programme/from patient) THEN the GP will be more likely to support their ongoing engagement.

3. Adherence
    d. Patient

IF there is a significant change (+/-) in patient condition (symptoms) THEN this may affect adherence (+/-).

IF there is a significant change in patient motivation (GP order vs recommendation/patient belief/alternative treatments) THEN this may affect adherence.

IF there is a significant change in patient enjoyment (social aspects) or fulfilment (condition needs/intervention need/goal attainment) THEN this may affect adherence.

IF the patient can continue to access the intervention (child care/transport/location) THEN this may affect adherence.
